# Temporal trends in occupational accidents involving exposure to biological material in Brazil, 2010 to 2016

**DOI:** 10.47626/1679-4435-2021-565

**Published:** 2021-04-30

**Authors:** Sâmea Cristina Santos Gomes, Thais Furtado Ferreira, Arlene de Jesus Mendes Caldas

**Affiliations:** 1 Programa de Pós-Graduação em Saúde Coletiva, Coordenação do Curso de Medicina, Centro de Ciências Sociais, Saúde e Tecnologia, Universidade Federal do Maranhão (UFMA), Imperatriz, MA, Brazil; 2 Programa de Pós-Graduação em Saúde Coletiva, Departamento de Enfermagem, UFMA, Pinheiro, MA, Brazil; 3 Programa de Pós-Graduação em Saúde Coletiva, Departamento de Enfermagem, UFMA, São Luís, MA, Brazil

**Keywords:** occupational accidents, biological material, health care workers, time-series study

## Abstract

**Introduction:**

Accidents involving biological material are the main cause of occupational exposure among health care professionals.

**Objectives:**

To analyze trends in the number of accidents involving exposure to biological material among health care workers in Brazil, in the period of 2010 to 2016.

**Methods:**

This was an ecological study based on secondary data on occupational accidents involving biological material reported to the Information System for Notifiable Diseases (Sistema Nacional de Agravos de Notificação). The data were analyzed using descriptive methods, followed by a calculation of incidence rates per 1,000 workers per year. Lastly, trend analysis was performed using Prais-Winsten regression.

**Results:**

A total of 243,621 accidents involving exposure to biological materials were reported among health professionals in the study period. The highest incidence rate (16.74 accidents per 1,000 workers per year) was observed in 2014. Regional analyses showed that Midwestern, South and Southeast Brazil had higher incidence rates than other regions of the country. At the state level, the highest rates were observed in Roraima, Rio Grande do Norte, Alagoas, Goiás, Minas Gerais, São Paulo, Paraná, and Santa Catarina. National incidence rates of occupational accidents with exposure to biological material in health care workers had a stable trend over the study period.

**Conclusions:**

In Brazil, health care workers are disproportionately affected by occupational accidents with exposure to biological material. The present findings, together with other indicators, cast doubt on the stability of these figures over time.

## INTRODUCTION

Accidents involving exposure to biological material (AEBM) are the main cause of occupational exposure among health care professionals.^1-4^ These incidents can be defined as occupational accidents resulting in physical injury and direct contact with blood or other bodily fluids, as a result of percutaneous punctures by needles and other sharp objects, or direct contact with compromised skin and/or mucosa.^2,5^ The figures associated with occupational accidents in the country and around the world, as well as the incidence of these accidents, raise concerns about the precariousness of working conditions and the ineffectiveness of workplace regulations, emphasizing the need to discuss the temporal trends of these data.^6^

A study performed in Massachusetts, in the United States, reported that 16,158 AEBM occurred between 2002 and 2007, representing a mean of 2,693 incidents per year.^7^ In Italy, 99,435 accidents were reported between 1994 and 2013.^8^ In Brazil, according to the Information System for Notifiable Diseases (Sistema Nacional de Agravos de Notificação; SINAN), a total of 203,709 cases of AEBM were reported between 2007 and 2013, of which 76.86% (156,572) involved health care professionals.^9^

Previous studies have used temporal trends in the incidence of occupational accidents to evaluate variables associated with this issue and predict the evolution of these phenomena in Brazil and around the world.^10-12^ A study by Almeida et al.^6^ identified a significant decreasing trend in the incidence of typical occupational accidents, overall accidents, and incidents resulting in death. The authors also noted that these findings can be at least partly attributed to underreporting.

Unfortunately, time-series studies of national data on AEBM among health care workers are scarce and geographically limited. Recent studies involving the time-series analysis of occupational accidents have been limited to typical and commuting accidents and overall prevalence rates, as determined by Accident Reporting Forms (CAT) or the Annual Social Security Statistical Bulletin (AEPS).^6,13^ Some studies are also limited to a particular city or region^14,15^ or focus on mortality and risk factors in Brazil.^6,16-18^

There is, as such, a lack of nationwide estimates and descriptions of the geographical distribution and temporal trends of these data, which could provide a basis for public policies directed at health care workers by reflecting the performance of existing initiatives to reduce the number of accidents and improve reporting.^6^ In light of these observations, and the relevance of issues associated with the frequency and reporting of AEBM, the aim of the present study was to analyze trends in the incidence of these accidents among health care workers in Brazil, based on reports received by the SINAN from 2010 to 2016.

## METHODS

An ecological time-series study was conducted using data on AEBM in health care workers in Brazil from 2010 to 2016. The study population consisted of all AEBM reported across the 26 states in Brazil and the Federal District, from January 1, 2010, to December 31, 2016.

AEBM were defined as any incident involving blood or other biological fluids, experienced by health care professionals during occupational activities with exposure to potentially infectious materials.^19^

Descriptive methods were used to calculate the frequency and percentage of occurrence of AEBM in health care professionals. Incidence coefficients were calculated using the following formula: incidence of AEBM among health professionals = number of cases of AEBM in health professionals/population of health professionals x 1,000. The population of health care workers was determined based on the estimated number of active professionals for every year between 2010 and 2016.

In order to compare the incidence of AEBM between health professionals in different regions and states, these values were standardized based on the population of health care workers in Brazil in 2010. Incidence rates for AEBM were also stratified by age group (≥ 18 years, 20 to 39 years, 40 to 59 years, 60 to 64 years, > 65 years) and gender (male and female).

Temporal trends were analyzed using a Prais-Winsten regression model, which corrects for serial correlation, or the similarity between the measurements of a given variable obtained at different time points.^17^ These results allowed for each trend to be classified as increasing, stable, or decreasing. The mean annual variation and 95% confidence interval (95%CI) was also calculated for each coefficient, with positive values considered indicative of an increase and negative values interpreted as a decrease. Rates that did not significantly differ from 0 (p > 0.05) were considered stable. The total variation for the period was calculated as the percentage difference between the incidence rates in 2010 and 2016.

Incidence rates and trends for the country as a whole, as well as each region and state, were calculated using STATA 14.0. In accordance with the requirements of National Health Council (Conselho Nacional de Saúde) Resolution No. 466/2012, this study was evaluated and approved by the Research Ethics Committee of the Hospital Universitário Presidente Dutra at the Universidade Federal do Maranhão (HU-UFMA), under protocol No. 2.039.925/2017.

## RESULTS

The SINAN received 331,603 reports of AEBM between 2010 to 2016. Health care workers were involved in 243,621 (73.42%) of these cases. During the study period, a mean of 34,803 incidents involving health professionals were reported every year, and 95 such incidents were reported per day.

The highest incidence of AEBM (16.84 accidents per 1,000 workers per year) was observed in 2014, while the lowest (14.01 per 1,000 workers per year) was recorded in 2010 (Table 1). In 2016, the highest incidence rate was observed in the state of Paraná (24.70 per 1,000 workers per year), and the lowest, in Paraíba (3.65 per 1,000 workers per year). In northern Brazil, the state with the highest incidence of AEBM was Roraima (16.99 per 1,000 workers per year), while in the Midwest, it was Goiás (22.29 per 1,000 workers per year). Lastly, in the Southeast, Minas Gerais was the state with the highest incidence of reported accidents in 2016 (16.20 per 1,000 workers per year).

**Table 1:** Incidence rates (per 1,000 workers) and annual percent change in occupational accidents involving exposure to biological materials, Brazil, regions and states, 2010-2016

Regions and states	2010	2011	2012	2013	2014	2015	2016	Annual % change (95%CI)	p-value*	Situation
North	6.0	7.07	9.80	10.96	12.33	11.55	10.44	100.97 (-0.003 to -0.087)	0.066	Stable
Rondônia	5.5	6.02	7.0	7.15	7.99	9.37	8.65	9.68 (0.032 to 0.047)	< 0.001	Increasing
Acre	1.9	1.21	1.81	1.92	3.63	4.90	8.37	32.74 (0.057 to 0.18)	0.005	Increasing
Amazonas	2.07	2.84	12.66	17.57	20.23	20.50	15.11	42.36 (-0.001 to 0.307)	0.005	Increasing
Roraima	17.66	17.13	17.24	20.03	17.17	17.69	16.99	-0.08 (-0.01 to 0.01)	0.932	Stable
Pará	3.73	4.76	4.67	4.23	5.8	5.04	5.21	8.25 (0.001 to 0.05)	0.005	Increasing
Amapá	8.42	7.81	8.82	15.05	12.07	9.79	14.99	9.19 (-0.001 to 0.07)	0.055	Stable
Tocantins	19.21	22.57	23.71	20.78	22.38	16.73	15.48	-4.45 ( -0.05 to 0.011)	0.160	Stable
										
Northeast	7.83	9.73	10.54	11.7	12.62	12.47	10.48	54.57 (0.008 to 0.054)	0.119	Stable
Maranhão	4.39	5.69	4.60	4.13	4.6	4.68	5.16	-0.04 (-0.02 to 0.02)	0.982	Stable
Piauí	2.74	5.46	5.83	6.19	7.39	9.67	4.91	-57.48 (-0.94 to 0.20)	0.156	Stable
Ceará	8.35	9.75	11.43	13.77	13.79	12.12	8.26	-35.72 (-0.76 to 0.45)	0.784	Stable
Rio Grande do Norte	14.69	17.53	16.63	16.85	17.21	19.57	17.14	2.58 (0.004 to 0.018)	0.010	Increasing
Paraíba	4.51	6.30	7.67	10.77	8.37	10.46	3.65	2.37 (-0.07 to 0.09)	0.773	Stable
Pernambuco	3.15	5.35	8.76	13.41	16.48	16.07	14.51	-71.4 (-0.91 to -0.17)	0.013	Decreasing
Alagoas	21.37	21.42	22.38	17.99	19.49	16.71	18.60	-4.41 (-0.02 to -0.01)	0.001	Decreasing
Sergipe	17.80	15.01	16.43	12.95	14.32	11.95	12.81	-5.58 (-0.027 to -0.021)	< 0.001	Decreasing
Bahia	7.73	10.71	10.22	11.57	12.63	12.39	10.83	-47.51 (-0.70 to 0.14)	0.148	Stable
										
Southeast	18.13	19.04	18.81	18.03	17.87	16.87	14.97	-30.60 (-0.02 to -0.001)	0.032	Decreasing
Minas Gerais	12.99	16.42	18.68	17.45	17.68	18.75	16.20	3.16 (-0.01 to 0.03)	0.218	Stable
Espírito Santo	9.77	11.56	10.77	11.24	15.92	14.87	12.88	6.68 (0.005 to 0.05)	0.024	Increasing
Rio de Janeiro	15.09	18.22	17.79	16.72	16.38	12.44	12.31	-4.89 (-0.05 to 0.006)	0.105	Stable
São Paulo	22.25	21.12	19.92	19.34	18.66	17.80	15.55	-4.92 (-0.026 to -0.017)	< 0.001	Decreasing
										
South	14.83	16.83	20.31	22.31	23.10	22.23	21.25	62.82 (-0.002 to -0.055)	0.067	Stable
Paraná	23.45	23.37	28.83	28.66	26.58	23.21	24.70	0.41 (-0.02 to 0.02)	0.854	Stable
Santa Catarina	15.49	18.18	21.50	27.21	29.25	25.99	22.60	7.00 (-0.018 to 0.07)	0.172	Stable
Rio Grande do Sul	5.56	8.80	10.57	12.74	16.02	19.00	17.14	-64.47 (-0.94 to 0.42)	0.066	Stable
										
Midwest	14.01	14.98	16.56	17.14	16.07	17.12	15.31	17.93 (-0.007 to 0.02)	0.254	Stable
Mato Grosso	11.24	12.93	15.21	13.14	18.57	19.85	16.67	-0.55 (-0.019 to 0.014)	0.733	Stable
Mato Grosso do Sul	13.58	18.71	17.16	16.64	11.98	13.34	10.27	1.68 (-0.013 to 0.027)	0.401	Stable
Goiás	19.67	17.54	22.05	25.49	21.07	22.84	22.29	-48.09 (-0.59 to 0.02)	0.064	Stable
Federal District	8.40	10.52	9.15	8.30	9.97	9.24	8.26	-0.97 (-0.018 to 0.01)	0.482	Stable
										
Brazil	14.01	15.32	16.25	16.56	16.84	16.24	14.52	17.66 (-0.008 to 0.02)	0.321	Stable

Source: Ministry of Health (Ministério da Saúde; MS)/General Directorate of Occupational Health (Coordenação Geral de Saúde do Trabalhador; CGSAT)/Information System for Notifiable Diseases (Sistema Nacional de Agravos de Notificação; SINAN) (2017); Ministry of Health (Ministério da Saúde; MS)/National Registry of Health Establishments (Cadastro Nacional de Estabelecimentos de Saúde; CNES) (2017).

95%CI = 95% confidence interval.

* Prais-Winsten regression (p < 0.05).

The analysis of temporal trends per region and state revealed significant variations in the extent to which health care professionals are affected by AEBM across the country.

While the overall incidence rate in northern Brazil was classified as stable, the states in the region showed significant variability, as shown in Table 1. Increasing trends were observed in Rondônia (9.68%), Acre (32.74%), Amazonas (42.36%) and Pará (8.25%), while rates in Roraima, Amapá and Tocantins appeared to be stable (Table 1).

The overall trend of the northeastern region was also classified as stable. Yet state-level analyses revealed an increasing trend in Rio Grande do Norte (2.58%), and decreases in Pernambuco (-71.4%), Alagoas (-4.41%) and Sergipe (-5.58%). All other states in the region showed stable incidence rates, reflecting the general trend observed in the Northeast (Table 1).

Southeastern Brazil showed decreasing incidence rates for AEBM among health professionals during the study period (-3.06%). However, the state of Espírito Santo actually evidenced an increase in these figures (6.68%). Minas Gerais and Rio de Janeiro remained stable, while the state of São Paulo, which reported the highest frequency of AEBM, displayed a decreasing trend during the study period (Table 1).

The southern and midwestern regions of the country also showed stable trends over time, at both regional and state levels (Table 1).

## DISCUSSION

It can be challenging to analyze AEBM in a country as large as Brazil. Therefore, our analyses were conducted at three different levels (nationwide, by region and by state) to reduce the area of analysis and allow for more precise observations. The present findings showed the relevance and impact of AEBM on health professionals, revealing the magnitude and trends of these incidents in Brazil, and in individual regions and states, from 2010 to 2016.

The present study revealed significant variation in incident coefficients throughout the country, revealing a heterogeneous distribution across the national territory. Similar observations regarding the incidence of AEBM from 2007 to 2010 were made in the Epidemiological Bulletin of the Universidade Federal da Bahia,^19^ published in 2011. However, the study in question included all occupations, and did not focus exclusively on health professionals.

It is possible that differences between regions and states are not entirely attributable to workers’ individual characteristics, and are instead caused by variations in the structure and formulation of labor laws, as well as the technical, economic, social, cultural and political aspects of their implementation.^6^

The time-series analysis performed in the present study showed that the incidence of AEBM in health care workers in Brazil followed a stable trend. A similar observation was made in a study conducted in Iran,^20^ where a time-series analysis of data for the period of 2000 to 2011 revealed a stable frequency of this type of accident. While other studies have demonstrated a decline in these rates from 1998 to 2008^6^ and 1997 to 2009,^15^ these studies focused on occupational accidents as a whole rather than AEBM in populations of health care workers.

In conclusion, despite the limitations of the data and the fact that underreporting is likely to have caused an underestimation of accident rates, the fact that these figures remained stable over the years suggests a need for prevention strategies targeting occupational health and safety, such as frequent safety inspections. Furthermore, despite the stability of incidence rates in the country as a whole, the number of accidents reported actually increased during the study period.

Regional comparisons of accident rates revealed significant variability in these figures, suggesting that the extent to which health care workers are affected by AEBM may vary depending on the region and state in which they live. Incidence rates in the northern, northeastern, midwestern and southern regions of Brazil remained stable over the study period, reflecting the overall national trend. In the South, however, these figures tended to decrease.

Given the variability in the temporal trends of AEBM between regions and states, each of these values was individually classified according to its overall tendency, based on the following categories: increasing, stable, and decreasing. The present study revealed a stable trend in the states of Roraima, Amapá, Tocantins, Maranhão, Piauí, Ceará, Paraíba, Bahia, Minas Gerais, Rio de Janeiro, Rio Grande do Sul, Paraná, Santa Catarina, Mato Grosso do Sul, Mato Grosso, and Goiás, as well as the Federal District. However, while the overall trends were stable, the increase in the number of accidents during the study period suggests that the risks to which health care workers were exposed did not decrease, and work conditions are unlikely to have improved.

A time-series study performed by Pereira & Torres^14^ in the state of Ceará based on data for the period of 2009 to 2012 reached a different conclusion than the present study, finding a decreasing trend in the incidence of AEBM (34.4%), with a mean of 6.7 accidents per 1000 health care workers. This trend may be related to the flexibility and deregulation of the labor market, and an increase in practices such as outsourcing. The lack of oversight of these activities, combined with a greater reliance on subcontractors, may have led to an increase in the frequency of occupational accidents. The distance between employers and employees in these circumstances may contribute to underreporting. While the investment in occupational health and safety may have increased in previous years, few studies have evaluated the performance of existing occupational health programs. The few investigations on the subject have focused on narrowly targeted programs addressing specific risk factors and injuries, and have also displayed significant methodological shortcomings.^6^

The states of Pernambuco, Alagoas, Sergipe and São Paulo trended toward a decrease in the rates of AEBM among health care workers. São Paulo accounts for the highest number of incidents in the country, and the decrease in cases from 2015 to 2016 may have driven the trend towards a decrease. The states of Pernambuco, Alagoas and Sergipe showed an increase in the number of registered health care workers, which may have caused the decrease in incidence rates in 2015 and 2016 relative to previous years.

According to Wünsch Filho,^10^ decreasing trends in occupational accidents may be associated with changes in the raw number of incidents (which does not depend on the size of the worker population) as well as incidence rates themselves (which represent a ratio of accidents to the number of workers, and may vary depending on the latter).

However, it is important to note that the absence or reduction of reports does not necessarily indicate that no accidents have taken place, and may actually reflect underreporting. Some studies^21-24^ suggest that over 50% of workers fail to report occupational accidents. The resulting bias in the epidemiological data on occupational accidents may prevent a deeper comprehension of this issue, especially when a significant percentage of workers are missing from official statistics.^25^

The underreporting of occupational accidents is influenced by several actors and factors, including the victims of non-fatal occupational accidents. Many workers may fail to identify an association between their accident and their occupation, in addition to being simply unaware of the need to report such incidents. As a result, many workers may not report AEBM, as they are unaware of how and when to do so.^25^

According to previous studies,^24-27^ additional reasons for the underreporting of occupational accidents include: fear of retaliation at work, perceiving the accident as an inherent part of their job, lack of awareness of reporting protocols, having no other occupation, and believing that reporting the accident will not solve the problem.

Underreporting can have a negative impact on the selection of priorities for prevention and protection measures targeting the health and safety of workers.^27^ Underreporting hampers the analysis, assessment, planning and implementation of effective public policies. Without knowing which sectors are most affected, where and how these incidents occur, their frequency and incidence rates, and other relevant variables, administrators are limited in their efforts to address this public health issue.^28^

In northern Brazil, accident rates trended toward an increase in the states of Rondônia, Acre, Amazonas and Pará. A similar tendency was observed in the state of Rio Grande do Norte, in northeastern Brazil, and the state of Espírito Santo in the Southeast. The state of Amazonas showed the highest growth rate in the region (42.74%).

Across all aforementioned states, there was a gradual increase in reports of AEBM, and in the number of registered professionals. However, the findings appear to demonstrate that the increase in the number of professionals was not followed by an improvement in working conditions, which may have contributed to the increased number of occupational accidents in these locations.

Other factors that may have contributed to the increase in the number of AEBM in Brazil are improvements in reporting and changes in the criteria used to define an incident as an occupational accident, especially from 2007 onward. That was the year social security benefits and compensation began to be offered to victims of occupational accidents and illnesses, even in the absence of an official report; in these cases, incidents were evaluated based on the victim’s occupational category, the Technical Epidemiological Nexus for Social Security (Nexo Técnico Epidemiológico Previdenciário; NTEP) or the Technical Nexus between Illnesses and Occupational Accidents (Nexo Técnico por Doença Equiparada a Acidente de Trabalho; NTDEAT).^29^ These data confirm the hypothesis that Brazil has a relatively high incidence of occupational accidents, especially since, as noted by the Ministry of Labor and Employment,^29^ “there is flagrant violation of laws, and an obvious need for preventive interventions.”

The main methodological limitation of the present study pertains to the quality of the data, which was collected from the SINAN, and therefore subject to varying levels of underreporting and significant heterogeneity across states and regions. Limitations regarding the rates calculated for states with a small population and high rates of underreporting were addressed by calculating rolling averages. The conclusions drawn in the present study must also be interpreted in light of the legislative changes made in 2007, which still do not appear to have been fully implemented, given the inconsistent reporting of AEBM by health care workers as a whole.

Strengths of this study include its contribution to the characterization of AEBM among health care workers, especially since previous studies on the topic have not focused specifically on this population. The present study also analyzed data at a national level rather than focusing on a particular region or state, so that its findings may contribute to decision making across different levels of implementation of accident prevention and management programs in the country. The time-series analysis of the incidence of AEBM among health care workers revealed that these rates displayed a stable trend between 2010 and 2016. However, the trends varied significantly between units of analysis, with some figures increasing, and others remaining stable or even decreasing, reflecting the economic and social diversity of Brazilian states.

We conclude that the incidence rates of AEBM among health care workers in Brazil show high regional variation, and that the stable trends observed at a national level do not reflect the situation in individual regions and states. This may be the result of underreporting and differences in the number of health care workers registered in each location. These findings should be considered during the organization of strategies to reduce the number of accidents and provide safety education, in order to ensure a positive impact on reporting and prevent the underestimation of this issue.
